# Clinical and microbiologic investigation of an expedited peri-implantitis dog model: an animal study

**DOI:** 10.1186/s12903-019-0837-y

**Published:** 2019-07-15

**Authors:** Wook Jin Seong, Georgios Kotsakis, Jong-Ki Huh, Soo Cheol Jeong, Ki Young Nam, Jong Ryul Kim, Young Cheul Heo, Hyeon-Cheol Kim, Lei Zhang, Michael D. Evans, Heather Conrad, Robert J. Schumacher

**Affiliations:** 1 Department of Restorative Sciences, School of Dentistry, University of Minnesota, Minneapolis, MN USA; 2 Department of Periodontics, School of Dentistry, UTHealth, San Antonio, TX USA; 3 Department of Oral & Maxillofacial Surgery, Yonsei University College of Dentistry, Seoul, South Korea; 4 Department of Restorative Dentistry, School of Dentistry, West Virginia University, Morgantown, WV USA; 5 Department of Dentistry, College of Medicine, Keimyung University, Daegu, South Korea; 6 Department of Endodontology, Maurice H. Kornberg School of Dentistry, Temple University, Philadelphia, PA USA; 7 Department of Conservative Dentistry, School of Dentistry, Pusan National University, Pusan, South Korea; 8 Biostatistical Design and Analysis Center, Clinical and Translational Science Institute, University of Minnesota, Minneapolis, MN USA; 9 Center for Translational Medicine, University of Minnesota, Minneapolis, MN USA

**Keywords:** Dental implant, Peri-implantitis, Expedited dog model

## Abstract

**Background:**

Animal studies are pivotal in allowing experimentation to identify efficacious treatment protocols for resolution of peri-implantitis. The purpose of this investigation was to characterize an expedited dog peri-implantitis model clinically, radiographically, and microbiologically.

**Methods:**

Eight hound dogs underwent extractions (week 0) and implant (3.3 × 8.5 mm) placement with simultaneous surgical defect creation and ligature placement for induction of peri-implantitis (week 10). Ligatures were replaced at 6 weeks (week 16) and removed after 9 weeks (week 19) when supporting bone loss involved approximately 50% of the peri-implant bone. Microbial samples from the defects and healthy control implant sites collected at week 19 were analyzed utilizing a microarray. Clinical measures of inflammation were obtained and radiographic bone loss was measured from periapical radiographs. Radiographic depth and width measurements of bony defect were repeated at weeks 10 (baseline), 16, and 19. Canonical analysis of principal coordinates was used to visualize overall differences in microbial abundance between peri-implantitis and healthy implants.

**Results:**

This accelerated disease protocol led to intrabony defect creation with a mean depth and width of 4.3 mm and 3.5 mm, respectively after 9 weeks of ligature placement. Microbial identification revealed 59 total bacteria in peri-implant sites, 21 of which were only present in peri-implant sites as compared to healthy controls. Overall microbial beta diversity (microbial between-sample compositional diversity) differed between peri-implantitis and healthy implants (*p* = 0.009).

**Conclusions:**

Within the limitations of this study, this protocol led to expedited generation of peri-implant defects with a microbial profile indicative of a shift to disease and defect patterns conducive to regenerative treatment. However, the possibility of potential spontaneous resolution of lesions due to the lack of a chronicity interval as compared to chronic disease models need to be further clarified and considered during preclinical peri-implantitis model selection.

**Electronic supplementary material:**

The online version of this article (10.1186/s12903-019-0837-y) contains supplementary material, which is available to authorized users.

## Background

Peri-implantitis is a bacterially induced inflammatory disease that affects functional implants. It is characterized by inflammation of the peri-implant mucosa and loss of supporting bone [1, 2]. The reported prevalence of peri-implantitis in the literature varies with studies reporting prevalence rates as low as 13% over an average of five and a half years of follow-up (187 patients) [3] up to 43% depending on the definition of disease [4]. As peri-implantitis may lead to implant failure, clinical researchers are interested in efforts to identify an appropriate treatment for peri-implantitis. Even though many approaches for treating peri-implantitis have been investigated, the consensus is that the most efficacious treatment modality has not yet been identified [5–8].

When reviewing the limited number of interventions that have shown positive results in controlling peri-implant inflammation, a clinical question arises: what is the true outcome of treatment? Ideally, treatment of peri-implantitis should lead to regeneration of the peri-implant bone that is in direct contact with the previously contaminated implant surface [9]. The term “re-osseointegration” has been coined to characterize true regeneration in the treatment of peri-implantitis [10]. It is reasonable to assume that the true outcome of peri-implant disease treatment studies should be bone-to-implant contact [11, 12]. Yet, due to ethical limitations, surrogates such as probing depths and/or per-implant attachment levels have to be utilized in human studies [7, 13]. Alternatively, the design of animal studies could allow the retrieval of histological cores for microscopy [14, 15].

Indeed animal studies have provided significant knowledge on the patterns of healing following peri-implantitis treatment and have also shown that radiographic bone fill and attachment loss may be inappropriate surrogates for re-osseointegration [9, 15]. Albeit their paramount significance, recently there has been a paucity of adequately powered animal studies to investigate the true outcome of peri-implantitis interventions. The high cost inherent to animal studies is undoubtedly an impeding factor for prospective researchers. Funding agencies and corporate sponsors award limited funds for research and in many instances human studies may be less costly alternatives. In comparison to human studies, animal studies bare additional costs for defect creation and healing time to better simulate human clinical conditions. The time required for “natural progression” [16] of peri-implant bone loss around induced peri-implant defects in animal models vastly increases the animal feeding and housing costs as well as surgical costs and maintenance personnel fees.

Therefore, the purpose of this investigation was to characterize an expedited dog peri-implantitis model clinically, radiographically, and microbiologically utilizing Human Oral Microbe Identification Microarray (HOMIM).

## Methods

**Fig. 1:**
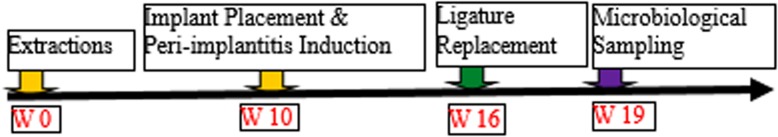
Schematic outline of the experiment

The study protocol (#1010A91692) for this study was approved by the Institutional Animal Care and Use Committee at the University of Minnesota. Animals were obtained through University of Minnesota Research Animal Resources (RAR). Animals were housed in RAR facilities and all surgeries were carried out at the surgical suites of Experimental Surgical Services of University of Minnesota. The University of Minnesota RAR adheres to the principles as stated in the Guide for the Care and Use of Laboratory Animals, National Academy Press, 2010. Study was carried out from February of 2011 and ended in June of 2012. Eight 1-year-old male Hound dogs with weights ranging from 25 to 33 kg underwent extractions, implant placement and ligature placement for induction of peri-implantitis utilizing an expedited approach. The schematic outline of the experiment is shown in Fig. 1.

### Defect generation

Experimental Surgical Services team of University of Minnesota prepared animals, induced and monitored anesthesia, and was in charge of recovery following their protocols. All dogs received a prophylactic antibiotic (Ceftiofur, 3 mg/kg IM) the evening before each early morning surgery for tooth extraction at baseline (W0) and implant placement after 10-week extraction healing (W10). A sedative (Acepromazine, 0.2 mg/kg IM) and analgesic (Buprenorphine 0.02 mg/kg IM) were administered before the induction of anesthesia. General anesthesia was induced by administering Propofol (2–6 mg/kg IV) and was maintained with Oxygen (2–4 L/min) and Isoflurane (1–3%). Four teeth in the mandible (left and right P4 and M1; 40 mm mesiodistal space in average) and two teeth in the maxilla (left and right P4; 22 mm in average) were extracted in each dog.

**Fig. 2:**
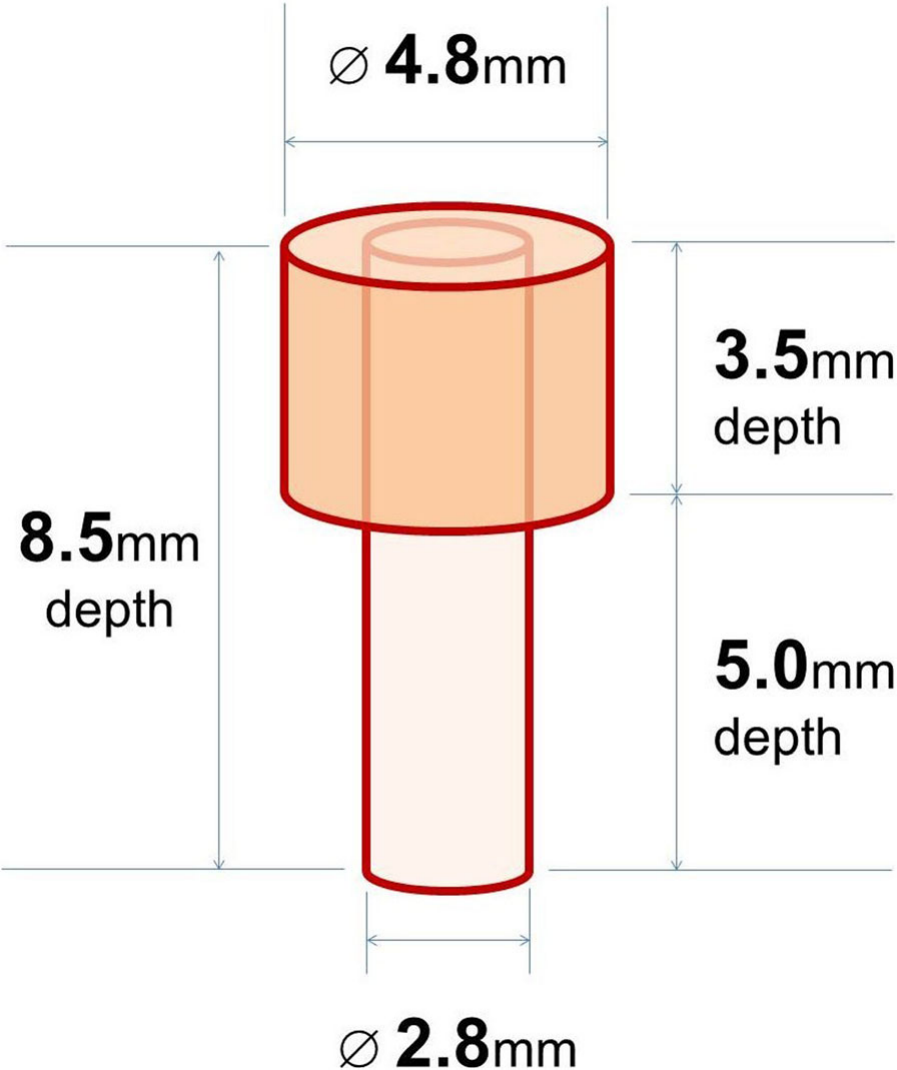
Illustration of the simulated peri-implantitis defect design (4.8 mm in diameter × 3.5 mm in depth) for 3.3 × 8.5 mm implant

Ten weeks after extractions (W10), a total of 10 identical 3.3 × 8.5 mm self-threading endosseous dental implants (PESF3308R, Dio Corp., Busan, Korea) with resorbable blast media (RBM) surfaces were placed in the mandible and maxilla of each dog. Four 3.3-mm diameter implants were placed in the maxilla. One implant was self-threaded in a 2.8/2.4 mm diameter osteotomy utilizing standard surgical protocol and served as “Healthy Implant control group (HI group)” and the remaining three implants were placed for a separate experiment (data not included). Si× 3.3 mm diameter implants were placed in the mandible in sites simulating peri-implantitis defects and designated as “Peri-Implantitis Implant group (PI group)”. The defects were created in two steps. In the first step, an osteotomy was prepared in the dog’s mandible per routine surgical protocol for the placement of a 3.3 × 8.5 mm implant utilizing a 2.8/2.4 mm final drill. Subsequently, the coronal 3.5 mm of the osteotomy were prepared with a 4.8 mm drill to facilitate the peri-implant defect formation. At the end of the preparation the implants were self-threaded with direct bone contact at the apical 5 mm of the osteotomy, while the coronal 3.5 mm had a 0.75 mm moat around the 3.3 mm diameter implant (Fig. 2).

Healing abutments (5 mm height) were connected to all maxillary and mandibular implants according to a one-stage implant protocol (non-submerged healing). Ligatures (Ultrapak™, Ultradent Products, Inc., South Jordan, UT) were placed simultaneously with the implant surgery and left in the defects to facilitate plaque accumulation and peri-implantitis induction in the coronal part of the implants while the apical 5 mm of implant were to achieve osseointegration from self-threading. Ligatures were replaced once at 16-week (W16), six weeks after the implant placement surgery and clinical pictures and radiographs were taken to monitor bony defect development. The new replacement ligatures were left in situ until week 19 (W19) so that significant bony defects (40–60% bone loss) were created to resemble defects encountered in advanced peri-implantitis cases [17].

**Fig. 3:**
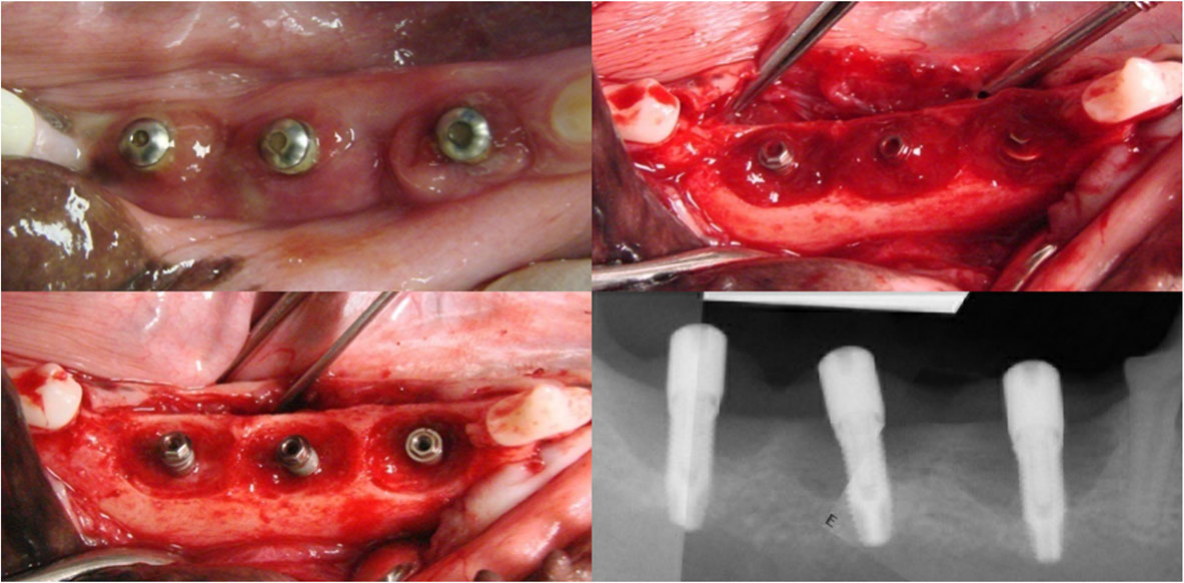
Clinical photographs and representative radiograph of the elicited defects taken at Week-19

Nine weeks after implant surgery (W19), all ligatures were removed and subgingival plaque samples were obtained from each implant for HOMIM. Clinical pictures and radiographs were obtained and bleeding on probing (BoP) was measured as an index of active peri-implant inflammation (Fig. 3). Following flap reflection, the configuration of the peri-implant defects was evaluated and clinical photographs were obtained. At this time interval peri-implantitis intervention surgeries were carried out for another research project (results not reported). Dogs were euthanized at weeks 23, 27, 31, and 71. A sedative (Acepromazine, 0.2 mg/kg IM) was given before the induction of anesthesia. Anesthesia was induced by administering 2–6 mg/kg Propofol IV to effect. Finally, Beuthanasia D solution 40 mg/kg IV was given for euthanasia.

### Evaluation

Clinical evaluation was performed at W19 after ligature removal. Bleeding on probing (BoP) was utilized to assess active peri-implant inflammation. Briefly, a periodontal probe (UNC-15) was utilized to probe the peri-implant defects circumferentially and bleeding on probing was assessed at 6 sites per implant as a dichotomous variable (i.e. bleeding, not bleeding). The configuration of defects after flap reflection was evaluated by an experienced examiner as horizontal, 1-wall, 2-wall, 3-wall, circumferential [18].

**Fig. 4:**
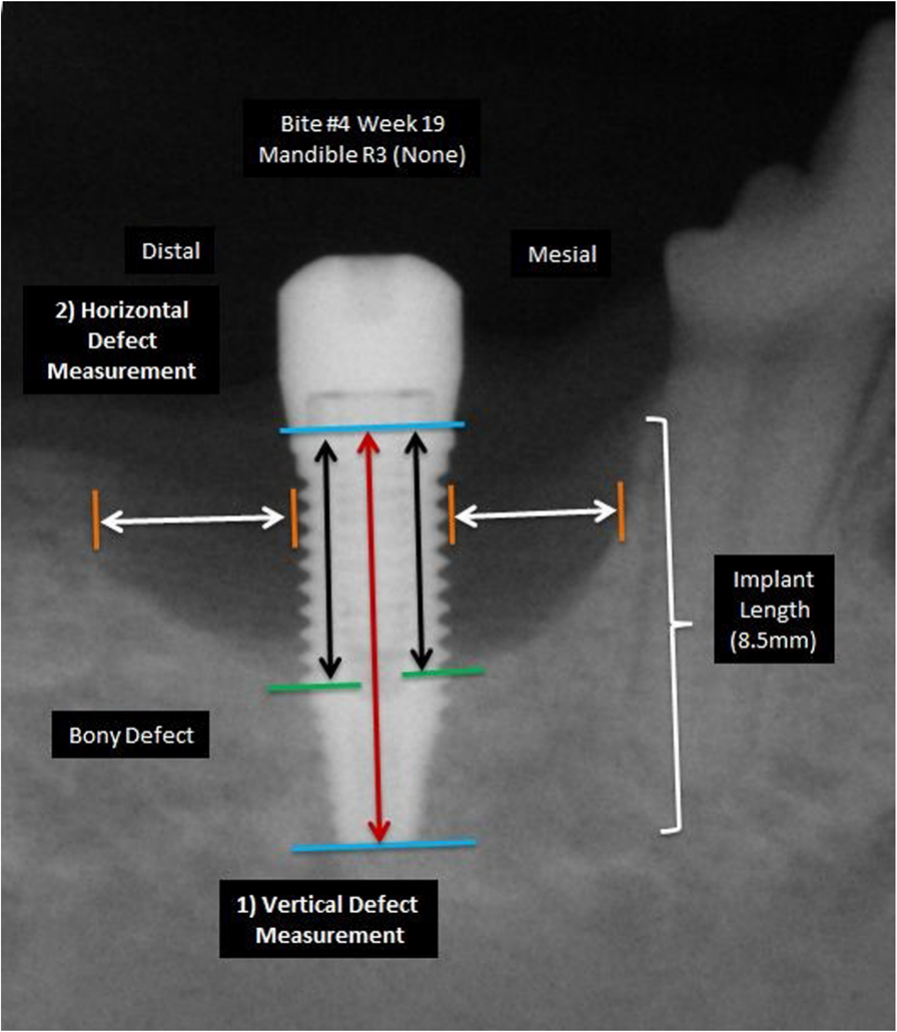
Vertical (depth, black arrows) and horizontal (width, white arrows) defect measurements

Radiographic evaluation was performed utilizing digital intra-oral radiographs (CDR, Schick technologies Inc., Long Beach, CA) that were obtained with a portable dental X-ray machine with the aid of an x-ray alignment device (XCP, Linn Dentsply, Elgin, IL) and the long-cone paralleling technique. The radiographs were further analysed to measure the defect size change around the implants at W10, W16 and W19, by measuring defect depth and width. Defect depth was defined as the linear distance from the implant platform to the depth of the peri-implant defect and width was defined as the linear distance from the threads of implant to the furthest edge of the defect (Fig. 4). All measurements were performed twice at 2 separate time points by a calibrated examiner using a specialized software that allowed use of the implant length as internal reference (ImageJ, NIH, Bethesda, MD). Intra-class correlation coefficient (ICC) was calculated to assess the examiner’s reliability between the two measurements.

Microbial sampling was performed using sterile plastic implant scalers. DNA was extracted from all samples using the recommended HOMIM protocol http://mim.forsyth.org) (on the day of collection. DNA extracts were frozen at − 80 °C and shipped to the HOMIM analysis core at the Forsyth Dental Center (Boston, MA). A detailed description of the HOMIM protocol including PCR primers, thermal cycling conditions, labelling, hybridization and normalization has been published previously [19]. The HOMIM arrays produce relative intensity values ranging from 0 to 5 (the minimum threshold for signal detection is equivalent to approx. 104 bacterial cells) [19]. This number provided a semi-quantitative estimate of the relative abundance of rDNA within each sample that hybridized with each probe.

### Statistical analyses

All analyses for radiographic bony defect sizes were performed with the SAS system (v. 9.3; SAS Institute, Cary, NC) and R version 3.5.2 (R Foundation for Statistical Computing, Vienna, Austria). The defect depth, defect width and defect depth percentage (defect depth/implant length 8.5 mm × 100) were summarized as mean ± SD at each time point for each group. The *p*-values for comparison of PI and HI groups at W16 and W19 were calculated from linear mixed models to account for within cluster correlation with dog considered as a random factor. Mixed-effects models were also employed to investigate the time effect among PI groups for defect depth, defect width and defect depth percentage. The estimated means ±SE were reported. Canonical analysis of principal coordinates (CAP) [20] was used to visualize overall differences in microbial between-sample compositional diversity (i.e. beta diversity) between peri-implantitis and healthy implants. Implant status (peri-implantitis or healthy) was used as the constraint variable, and the association of microbial abundance and implant status was assessed using permutation testing, by permuting the implant status labels 1000 times and calculating the proportion of permutations where the prediction (of implant status by microbial abundance) accuracy exceeded that of the non-permuted data to obtain a *p*-value.

## Results

During the extraction procedure (W0) Dog #1 passed away due to anesthesia complication and thus was excluded from the analysis. The data from one maxillary healthy implant (HI) as a control and 6 mandibular peri-implantitis implants (PI) per dog were reported from Dogs #2–8.

All implants exhibited 100% BoP at W19. Also, clinical evaluation performed after flap elevation revealed that this model led to the generation of circumferential bony defects (Schwarz Class 1e) [18] in most cases.

**Fig. 5:**
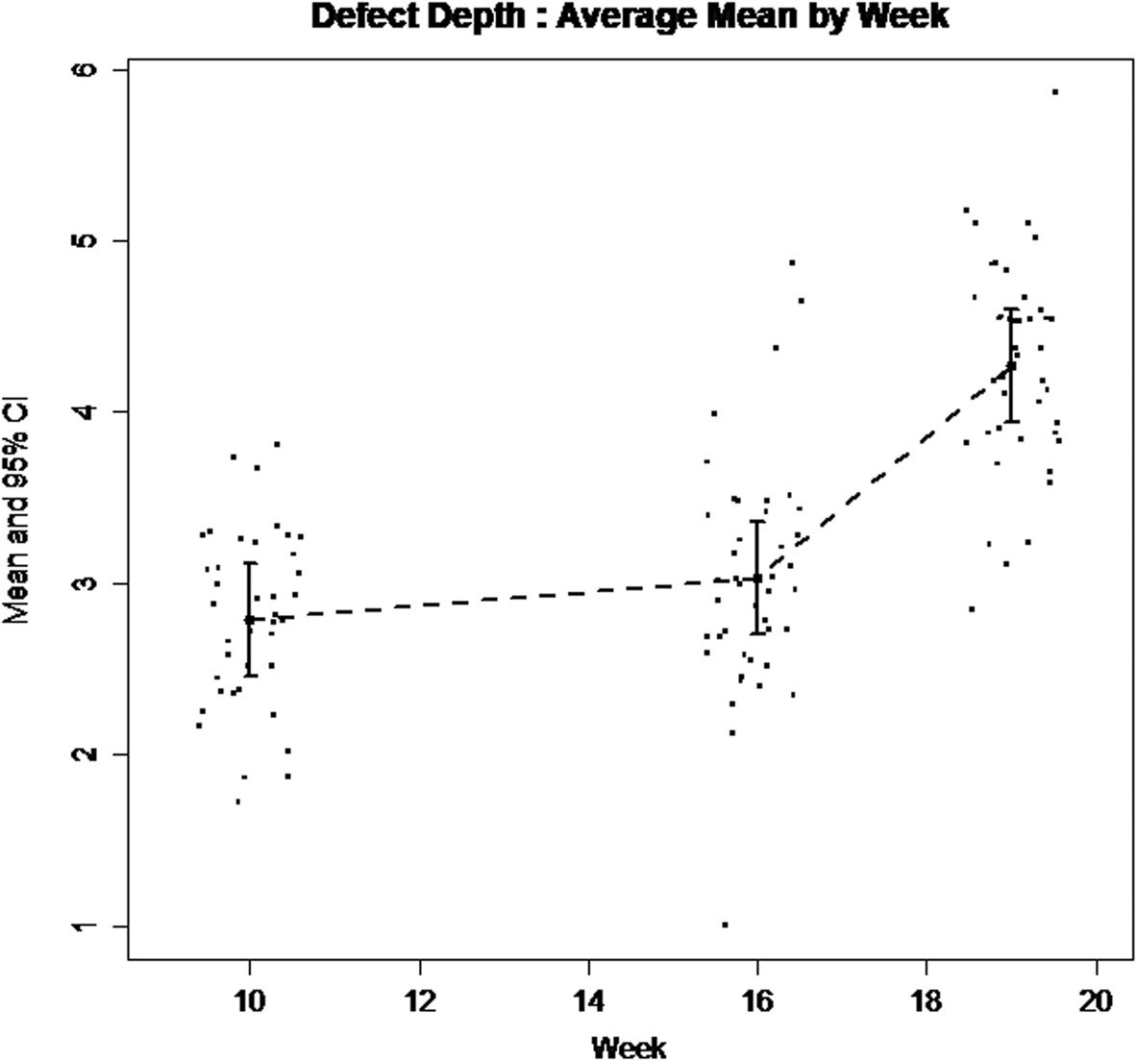
Defect depth change (mm) of the Peri-implantitis Implant group over the time (At Week-10 baseline, 3.5 mm deep and 4.8 mm wide defect was created surgically around 3.3 mm diameter implant leaving 0.75 mm wide moat around)

**Fig. 6:**
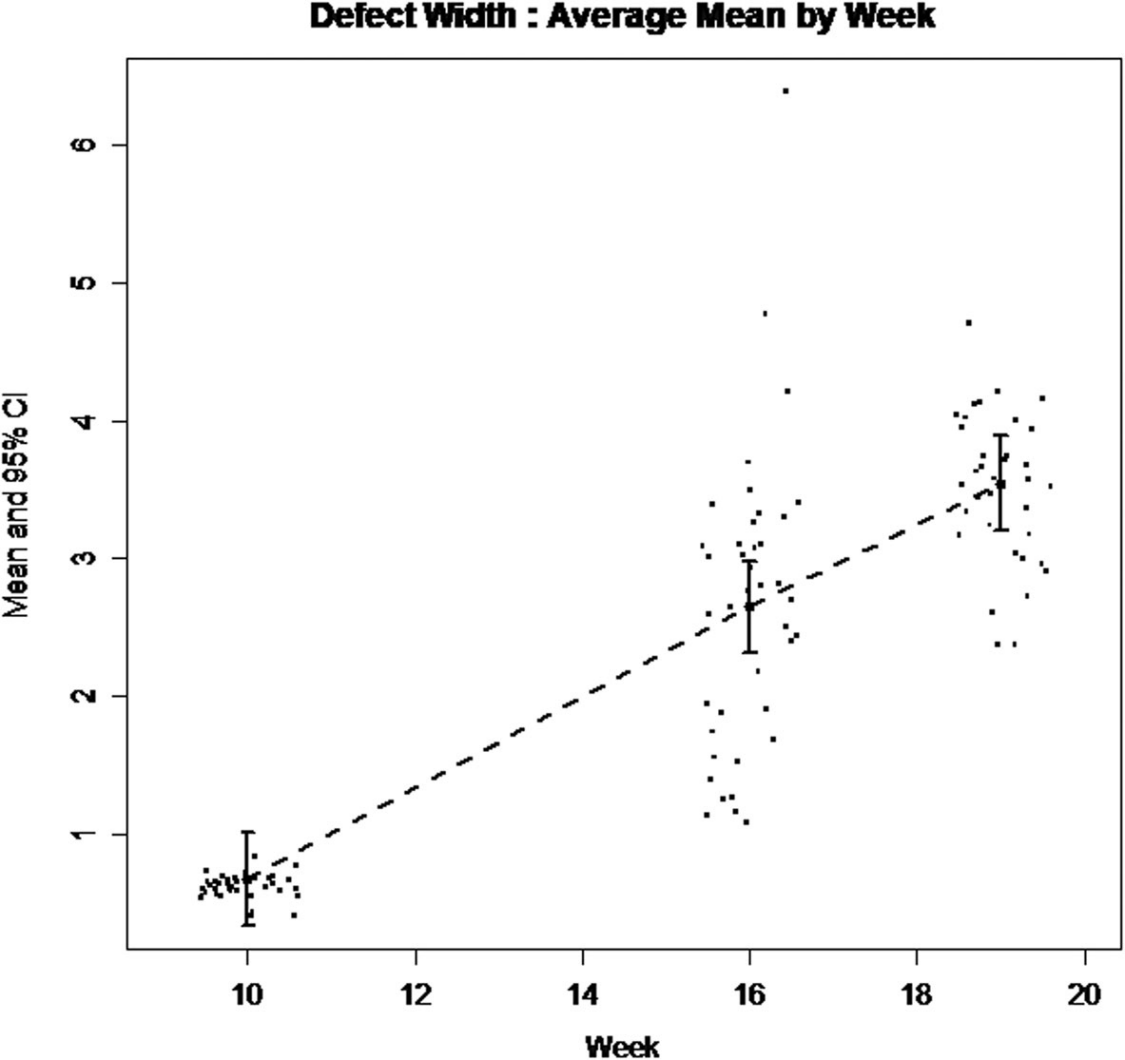
Defect width change (mm) of the Peri-implantitis Implant group over the time (At Week-10 baseline, 3.5 mm deep and 4.8 mm wide defect was created surgically around 3.3 mm diameter implant leaving 0.75 mm wide moat around)

Descriptive statistics on defect depth, defect width, and defect depth percentage measured from radiographs of W10, W16, and W19 are presented in Table 1. Defect size was significantly different between HI and PI groups at all 3 time points. Table 2 reports bony defect depth, width, and depth percentage estimates (SE) and *p*-values for the time effect. Pairwise comparison is presented in Table 3. Both defect depth and width significantly increased from the time of surgical defect creation and ligature placement (W10) to week 16, by 0.24 mm (*p* = .04) and 1.98 mm (*p* < .001), respectively. During the remaining three weeks from ligature replacement (W16) through week 19, a highly significant increase in defect depth and width was noted, 1.24 mm (p < .001) and 0.89 mm (p < .001), respectively (Figs. 5 and 6). Intra-correlation coefficient (ICC) assessing the reliability of two separate time defect measurements ranged from 0.77 to 0.95 (Table 4). The 100% of the repeat measurements were within 1 mm of the initial measurements.

**Table 1:** Descriptive statistics on defect depth, defect depth ratio, defect width and *P*-values between Healthy Implant and Peri-Implantitis implant groups

Week	Stats	Healthy Implant (*n* = 7)			Peri-Implantitis Implant (*n* = 42)			P-values		
		Defect Depth (mm)	Defect Width (mm)	Defect Depth %	Defect Depth (mm)	Defect Width (mm)	Defect Depth %	Defect Depth (mm)	Defect Width (mm)	Defect Depth %
W10	n	7	7	7	40	37	40			
	Median	0	0	0	2.80	0.63	32.91			
	Mean (SD)	0	0	0	2.77 (0.53)	0.62 (0.09)	32.59 (6.25)			
	(Min, Max)	(0, 0)	(0, 0)	(0, 0)	(1.73, 3.81)	(0.42, 0.84)	(20.29, 44.82)			
	Estimate (SE)	0 (0)	0 (0)	0 (0)	2.78 (0.17)	0.62 (0.02)	32.73 (1.97)	< 0.001	< 0.001	< 0.001
W16	n	4	3	4	42	40	42			
	Median	0.81	0.52	9.49	2.98	2.73	35.01			
	Mean (SD)	0.80 (0.22)	0.90 (0.72)	9.46 (2.61)	3.03 (0.68)	2.65 (1.07)	36.62 (8.04)			
	(Min, Max)	(0.54, 1.06)	(0.45, 1.73)	(6.38, 12.47)	(1.01, 4.87)	(1.09, 6.38)	(11.82, 57.24)			
	Estimate (SE)	0.85 (0.33)	0.72 (0.62)	9.98 (3.91)	3.03 (0.14)	2.65 (0.21)	35.62 (1.60)	< 0.001	0.004	< 0.001
W19	n	3	2	3	42	35	42			
	Median	1.51	0.73	17.76	4.35	3.57	51.13			
	Mean (SD)	1.40 (0.46)	0.73 (0.18)	16.42 (5.41)	4.27 (0.61)	3.51 (0.54)	50.26 (7.19)			
	(Min, Max)	(0.89, 1.79)	(0.61, 0.86)	(10.47, 21.03)	(2.84, 5.86)	(2.38, 4.71)	(33.44, 68.97)			
	Estimate (SE)	1.47 (0.34)	0.48 (0.32)	17.34 (3.96)	4.27 (0.14)	3.52 (0.17)	50.26 (1.69)	< 0.001	< 0.001	< 0.001

**Table 2:** Bony defect Estimate (SE) and p-value for time effect

Variables	Estimate (SE)			*P*-value
	Week 10	Week 16	Week 19	
Defect Depth (mm)	2.79 (0.15)	3.03 (0.15)	4.27 (0.15)	< .001
Defect Width (mm)	0.68 (0.15)	2.65 (0.15)	3.55 (0.16)	< .001
Defect Depth %	32.76 (1.73)	35.62 (1.71)	50.26 (1.71)	< .001

**Table 3:** Pairwise comparison of different time points

Variables	Difference (SE) and *P*-value		
	Week 10 vs. 16	Week 10 vs. 19	Week 16 vs. 19
Defect Depth (mm)	0.2425 (0.1154), *p* = .0388	1.4869 (0.1154), *p* < .0001	1.2444 (0.1138). *p* < .0001
Defect Width (mm)	1.9771 (0.1369), *p* < .0001	2.8690 (0.1418), *p* < .0001	0.8919 (0.1400), *p* < .0001
Defect Percentage	2.8531 (1.3581), *p* = .0388	17.4932 (1.3581), *p* < .0001	14.6401 (1.3393), *p* < .0001

**Table 4:** Intra-correlation coefficient (ICC) calculated to assess the examiner reliability at two separate time points for measurements

Variables	ICC
Defect depth – Mesial	0.93
Defect depth – Distal	0.95
Defect width – Mesial	0.83
Defect width – Distal	0.77

**Fig. 7:**
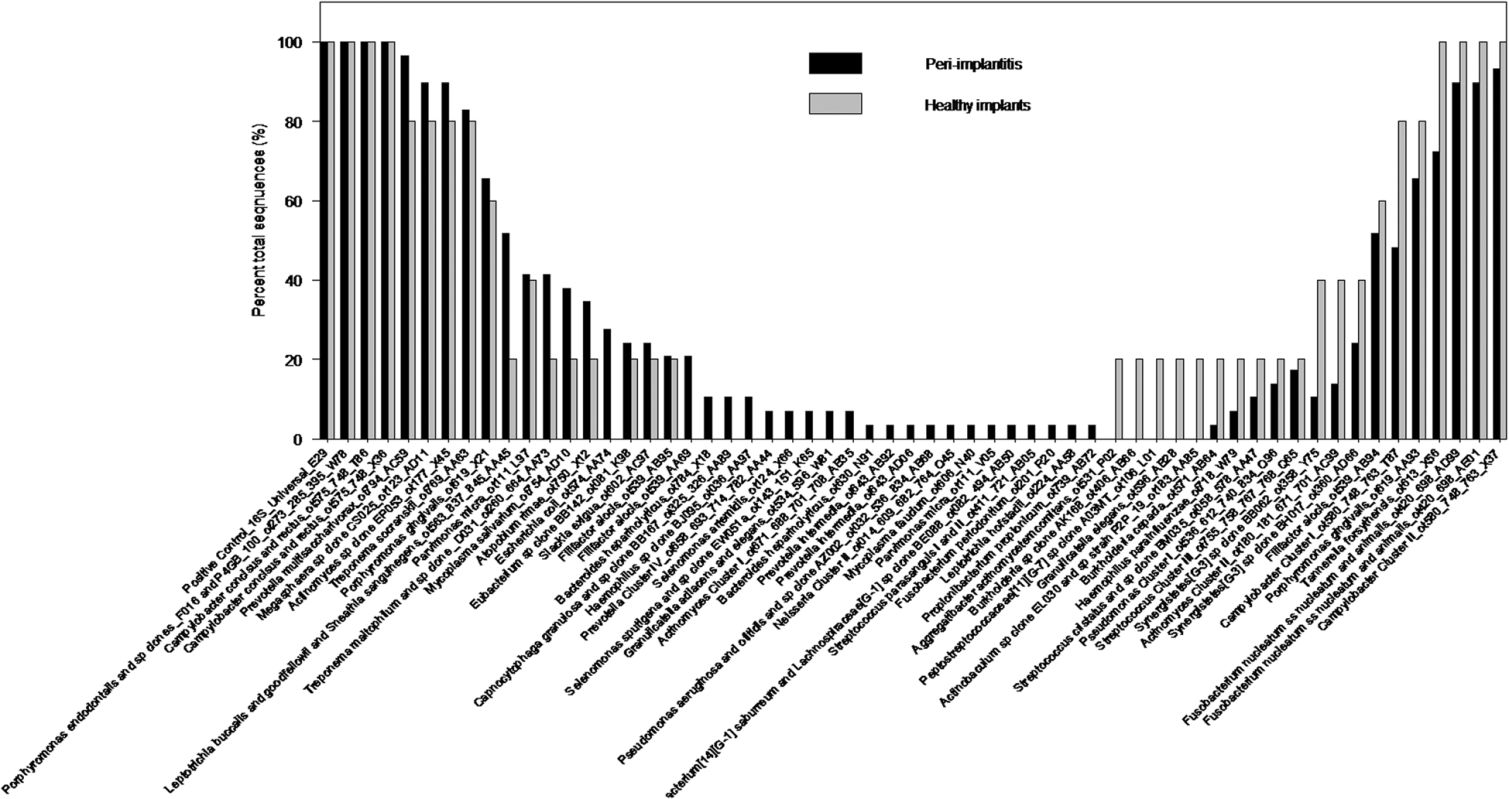
The intensity is dichotomized as presence (1, 2, 3, 4, and 5) and absence (0). The percent of presence is plotted by groups and bacteria types. There are 59 bacteria in total which are either present in Peri-implantitis Implant or Healthy Implant groups

**Fig. 8:**
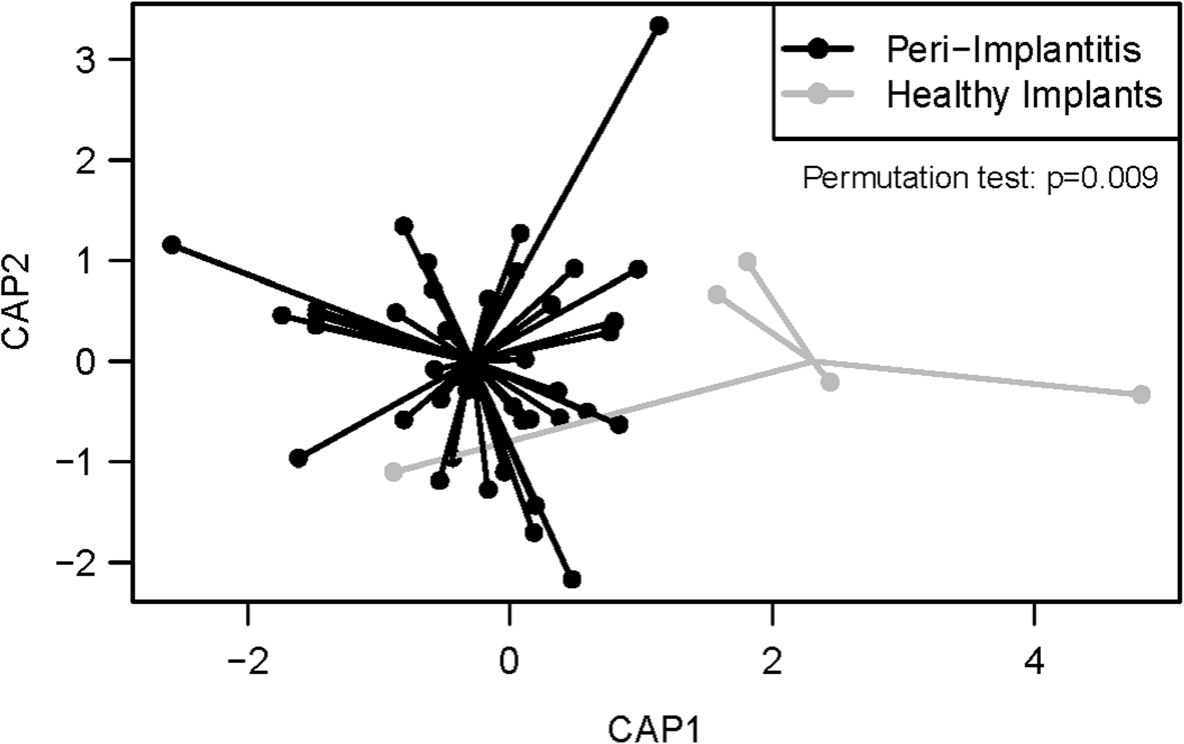
Canonical analysis of principal coordinates (CAP) comparing microbial abundances across all HOMIM probes between Peri-implantitis Implants and Healthy Implant groups. Axes represent first and second principal coordinates based on Euclidean dissimilarity in HOMIM probe intensities between samples

Microbial identification results per group (PI and HI) are presented in Fig. 7. There were 59 total bacterial taxa and 21 of them were present only in the PI group, while only 4 were present only in the HI group. Table 5 lists the 21 oral taxa that were unique to the PI group, which indicates a shift in the composition of the submucosal microflora in peri-implantitis implant sites as compared to healthy implant sites. Canonical analysis of principal coordinates (CAP) comparing microbial abundances across all HOMIM probes between peri-implantitis and healthy implants indicated that implant status was significantly associated with microbial composition (*p* = .009) (Fig. 8).

**Table 5:** Twenty one oral taxa present only in PI (Peri-implantitis Implant) group

ID	Bacteria Name
40	Filifactor alocis_ot539_AA69
3	Bacteroides heparinolyticus_ot784_X18
4	Capnocytophaga granulosa and sp. clone BB167_ot325_326_AA89
16	Haemophilus sp. clone BJ095_ot036_AA97
11	Prevotella Cluster IV_ot658_693_714_782_AA44
27	Selenomonas artemidis_ot124_X66
28	Selenomonas sputigena and sp. clone EW051a_ot143_151_K65
46	Granulicatella adiacens and elegans_ot534_596_W81
67	Actinomyces Cluster I_ot671_688_701_708_AB35
2	Bacteroides heparinolyticus_ot630_N91
8	Prevotella intermedia_ot643_AB92
9	Prevotella intermedia_ot643_AD06
17	*Pseudomonas aeruginosa* and otitidis and sp. clone AZ002_ot032_536_834_AB68
25	Neisseria Cluster II_ot014_609_682_764_O45
31	Mycoplasma faucium_ot606_N40
36	Parvimonas micra_ot111_V05
38	Eubacterium [14][G-1] saburreum and Lachnospiraceae [G-1] sp. clone BE088_ot082_494_AB50
51	Streptococcus parasanguis I and II_ot411_721_AB05
55	Fusobacterium periodontium_ot201_R20
58	Leptotrichia hofstadii_ot224_AA58
69	Propionibacterium propionicum_ot739_AB72

## Discussion

In the present study we explored the utilization of an expedited in vivo model for the generation of peri-implant defects. The proposed model was an acute trauma model that consistently led to the formation of intrabony defects (1e according to Schwarz’s classification [18]) with a mean depth and width of 4.3 mm and 3.5 mm, respectively, after 9 weeks of ligature placement. These defects accounted for approximately 50% of the total implant length. The fundamental difference between this expedited, acute-trauma model and previous ligature-induced peri-implantitis models is the combination of surgical defect creation at the time of implant placement followed by ligature-facilitated bone loss.

In the majority of previous studies, the most commonly utilized approach for eliciting creation of peri-implant bone loss around implants is based on the original work of Lindhe et al. that borrowed concepts from animal models of periodontitis and implemented them in peri-implantitis research [21]. The core of these ligature-induced peri-implantitis models was the placement of silk or cotton ligatures in the peri-implant sulci [21, 22]. Martins et al. [16] have debated that the ligature acts as a foreign body in the peri-implant sulcus, thus does not accurately mimic the progression of disease in humans [16]. To better simulate a “naturally-occurring” model of disease progression most researchers employ plaque accumulation periods of varying duration following ligature removal [18]. These periods of spontaneous progression have been found to be associated with cellular inflammatory infiltrates in the peri-implant tissues and with crater-shaped intrabony defects resembling human periodontitis [16, 18, 22–24].

In these “spontaneous progression” models the initiation of peri-implant inflammation occurs by means of submucosal placement of a ligature [22–24]. On the contrary, in acute disease models the defect is initiated surgically [10, 25]. This approach, as presented in our model, allows researchers to bypass the healing period after implant placement that averages 13 weeks in published studies and minimizes the active ligature-related breakdown period to 9 weeks. In the present study, the increase in defect depth seemed to be ligature-related, while the change in defect width demonstrated a linear pattern with time (Figs. 5 and 6). The event of ligature placement and replacement had a marked effect on the bone directly apically to the ligature (Fig. 5), but it did not directly affect the defect width that kept progressing linearly with time (Fig. 6). This may imply that a significant component of ligature-associated bone loss may be attributable to contact inflammation versus chronic. Notably, even though the ligature was placed concurrently with the implant insertion in the present study, none of the placed implants failed for a 100% implant integration rate that verifies the feasibility of the presented technique.

Overall, when comparing the experimental time required in our study from implant placement to formation of peri-implant defects to that in the model of Zitzmann et al. [22], approximately 20 months of animal stocking time were saved. That constitutes a tremendous financial benefit of the model presented herein.

Nonetheless, the presented model has limitations that must be weighed against the gain in animal stocking time. The main limitation of the presented model is the potential for spontaneous regression of the defects since they represent acute trauma situations. Such a regression could give inflated estimates on the outcomes of regenerative approaches or dilute the effect size in comparative regenerative studies. On the other hand, ligature-induced models have been extensively evaluated and have been shown to maintain the generated defects devoid of spontaneous regeneration following ligature removal [22]. To compensate for the potential for spontaneous regression in acute disease models, the use of appropriate control sites should be carefully planned in the study design phase. In addition, the histopathological features of spontaneous regression models seem to resemble the inflammatory cell infiltrate obtained from human biopsies [25]. On the other hand, the histopathological features of accelerated models have not been described. After all, a direct comparison of treatment response with a classic spontaneous progression model is needed because it is unknown if expedited model is going to have spontaneous healing because of the acute infection model used.

Nonetheless, the microbial composition of the peri-implant plaque samples in our model was characterized utilizing a microarray that allowed the detection of more than 200 distinct oral taxa [26]. Results showed a total of 59 oral taxa identified in the experimental peri-implant sulci with 21 oral taxa being unique to the peri-implantitis implants as compared to healthy implant controls. These included genera that are known to be associated with peri-implantitis, such as Actinomyces, Filifactor, Propionibacterium, Prevotella, Parvimonas, and Streptococcus [27–29]. This finding of a microbial shift towards a peri-implant pathogenic microbiota may indicate that despite the absence of a spontaneous progression period, the peri-implant defects from this model were representative of chronic human peri-implant defects from a microbiological perspective. Although HOMIM is a molecular identification approach using 16S rRNA it is still limited in that it is not an open ended method such as next 16S DNA sequencing approach.

In summary, the salient point of the proposed expedited model of peri-implant defects is the timeliness of generation of an appropriately sized defect by means of surgical facilitation of defect initiation. It was shown that this model led to the formation of peri-implant defects that allow testing of regenerative peri-implant protocols with no implant failures occurring in this study. Therefore, this model has the potential to allow researchers to study the treatment of peri-implantitis without the cost or time burden associated with previously reported models. However, use of this model requires understanding of its limitations; this model should be further investigated to eliminate any concerns with spontaneous defect regeneration and to characterize the histopathological characteristics of the defects.

## Conclusions

We characterized an expedited in vivo model for induced peri-implant defects around implants to be used in assessing peri-implantitis treatment strategies. The microbiota associated with these defects was diverse and included oral taxa that at least on the genus level resemble oral taxa frequently encountered in human peri-implantitis. Further, the configuration of the peri-implant defects consistently demonstrated an intrabony component. This acute disease model may be a cost- and time-effective alternative to the current standard of spontaneous progression peri-implantitis models. Nonetheless, comparative studies are warranted to evaluate the potential of this expedited approach for spontaneous healing that may bias study results.

## Additional files

Additional file 1: Bone Defect Measurement Data. (XLSX 240 kb)

Additional file 2: HOMIM Data (Human Oral Microbe Identification Microarray). (XLSX 79 kb)

## Data Availability

All data generated or analyzed during this study are included in this published article and its Additional files 1 and 2.

## Abbreviations

BoP: Bleeding on Probing
CAP: Canonical analysis of principal coordinates
DNA: DeoxyriboNucleic Acid
HI: Healthy Implant
HOMIM: Human Oral Microbe Identification Microarray
ICC: Intra-class correlation coefficient
IM: Intramuscular
IV: Intravenous
L: Liter
M1: first molar
min: minute
P4: 4th premolar
PCR: Polymerase Chain Reaction
PI: Peri-implantitis Implant
RAR: Research Animal Resources
RBM: Resorbable blast media
rDNA: ribosomal DNA
W: Week
